# A New Recycling Technology to Produce Premixed Thermal Insulating Mortars from Polyurethane Waste Foams

**DOI:** 10.3390/polym17162233

**Published:** 2025-08-17

**Authors:** Antonis Kountouris, Kypros Efstathiou, Nikolaos Kostoglou, Dimitrios Manolakos, Claus Rebholz

**Affiliations:** 1 School of Mechanical Engineering, National Technical University of Athens, 15780 Athens, Greece; manolako@central.ntua.gr; 2 Department of Mechanical and Manufacturing Engineering, University of Cyprus, 2109 Nicosia, Cyprus; 3 Nicolaides & Kountouris Metal Company Ltd., 2045 Nicosia, Cyprus; kypros.efstathiou@gmail.com; 4 Department of Materials Science, Montanuniversität Leoben, 8700 Leoben, Austria; nikolaos.kostoglou@unileoben.ac.at

**Keywords:** plastic waste, polyurethane, recycling, lightweight mortar, premixed mortar, thermal insulation materials, compressive strength, building materials, sustainable construction, pilot production

## Abstract

The increasing demand for sustainable construction materials has driven research into the reuse of plastic waste for renewable building applications. This study introduces a new lightweight insulating mortar for floor and roof systems, utilizing recycled rigid polyurethane (PU) foam as the primary aggregate. The binder mainly consists of Portland cement, with no added sand, and includes minor additives to enhance mechanical, physical, and thermal properties. Initial tests demonstrated that key performance metrics—density, compressive strength, and thermal conductivity—are significantly influenced by the PU content. As the proportion of PU increased, all three parameters decreased. The optimized formulation, comprising 92.25 vol.% PU foam, 6.75 vol.% cement, and 1 vol.% additives, achieved a low bulk density of 420 kg/m^3^, a compressive strength of 1 MPa, and a thermal conductivity of 0.07 W/m·K. A pilot-scale production system with a capacity of 1500 L/h (equivalent to 20 bags of 75 L) was subsequently designed, implemented, and validated. These findings underscore the potential of PU-based lightweight insulating mortars to reduce environmental impact and support the development of sustainable construction practices globally.

## 1. Introduction

The production of plastics, widely regarded as one of the most significant innovations of the 20th century, has surged in recent decades: it grew from around two million tonnes (Mt) in 1950 [1] to over 400 Mt by 2022 [2]. This figure is projected to double to 800 Mt by 2050 [1], primarily driven by economic development, population growth, and urbanization. Of this global production, around 5% is dedicated to polyurethane (PU), the sixth most widely used polymer worldwide [3], predominantly in the form of flexible (36%) and rigid (32%) foams [4].

**Figure 1:**
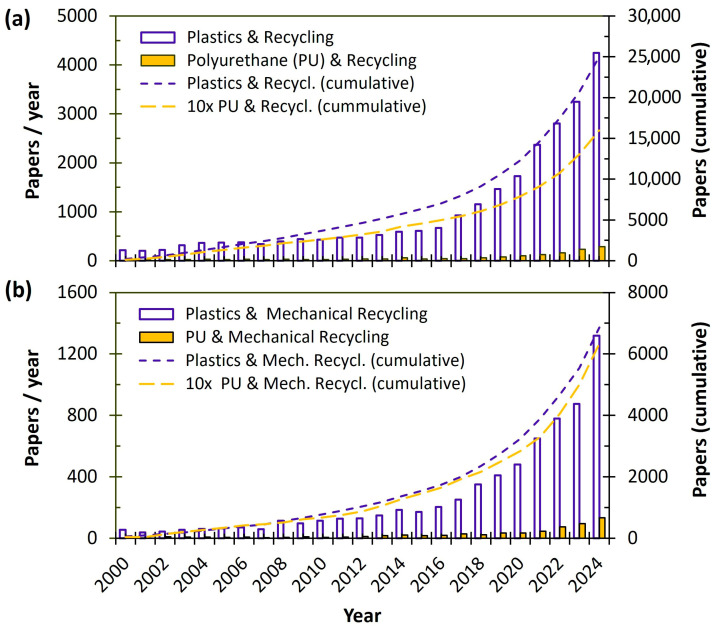
Search results within article title, abstract, and keywords (Scopus, February 2025) for the annual and cumulative numbers of publications (from 2000 to 2024) in peer-reviewed journals, including (**a**) Plastics & Recycling and Polyurethane & Recycling and (**b**) Plastics & Mechanical Recycling and Polyurethane & Mechanical Recycling.

Plastic waste has become a serious environmental threat, impacting both ecosystems and human health, and has thus gathered increasing attention [1]. In a very recent study, Dokl et al. [5] projected global plastic consumption and end-of-life pathways (landfilling, incineration, recycling, mismanagement, and littering) from 2000 to 2050 using an optimization-based regression model. Their analysis indicates that global plastic use may nearly double from 2020 to 2050, with landfilling remaining the dominant disposal method in several regions, particularly in Canada and the USA. In contrast, the European Union is expected to reduce landfilling to 16%, largely due to stringent regulatory measures. However, despite recent efforts to boost recycling rates in Europe, more than 70% of plastic waste in Cyprus, Greece and Malta was still sent to landfills in 2020 [6]. To avoid dumping plastic waste into landfills (still the most common way for PU [4,7,8]) or incinerating it for energy recovery, the end-of-life recovery and recycling of plastics, especially PU, has been a major focus in recent years. Figure 1a illustrates the rising number of peer-reviewed journal publications on this topic over the last 25 years, with approximately 25,000 papers published on plastics and recycling, and about 1600 on PU and recycling. Several recent review papers [3,4,7,8] have explored PU recycling methods, highlighting mechanical recycling (through grinding) and chemical recycling (mainly via glycolysis reactions) as the most promising approaches [4]. As shown in Figure 1b, approximately 600 papers have been published on PU and mechanical recycling from 2000 to 2024, compared to around 6900 papers on plastics and mechanical recycling during the same period.

The growing interest in sustainable construction materials has intensified research into the reuse of plastic waste as a raw material for renewable building products. Over the past few years, several review papers have been published, offering comprehensive overviews of plastic waste utilization in construction applications, particularly in cementitious composites [9,10,11,12]. In addition, several recent reviews focus specifically on the incorporation of PU waste in construction and building material composites [13,14,15].

The incorporation of rigid PU waste into cementitious composites not only diverts plastic waste from landfills but also reduces reliance on conventional materials such as aggregates and sand, thereby conserving natural resources and mitigating the environmental impacts associated with their extraction and processing. Characterization of five rigid PU waste types sourced from refrigerators and the automotive industry further demonstrated that all are suitable for use in the production of new construction materials [16].

Owing to the lower density of PU (1.05 g/cm^3^ [17]) compared to conventional aggregates such as sand and stone, its use leads to a significant reduction in the overall density of cementitious composites, resulting in lighter structural elements. Furthermore, the low thermal conductivity of PU (0.58 W/m·K [17]) contributes to reduced thermal conductivity in the composite material, thereby enhancing its thermal insulation performance. However, as observed with various types of plastic waste, the incorporation of plastic aggregates typically comes at the expense of reduced compressive strength in concrete and cement mortars [10,18].

The use of rigid PU waste in lightweight cement and mortar mixtures has been the focus of several studies. Notably, Mounanga et al. [19] pioneered the development of lightweight concrete, incorporating 13.1–33.7 vol.% of rigid PU waste (≤10 mm particle size), in 2008. This research was later expanded to include higher PU content (34–45 vol.%) and coarser particles (8–20 mm) [20]. Most recently, Rooban Kumar and Senthil Pandian [21] reported the replacement of course aggregates with 0–40% volume fraction of rigid PU waste (4.75–20 mm particle size) in concrete in an experimental study with ecological and cost assessment.

Lightweight mortars incorporating recycled PU foam particles were first introduced by Gadea et al. in 2010 [22]. In this initial study, sand was partially replaced with ground PU particles (0–4 mm), resulting in improved workability. A subsequent study by the same group employed smaller PU particles (0–1 mm) using the same methodology [23]. In a later investigation, the authors demonstrated that cement mortars with 50% and 60% sand substitution using shredded rigid PU particles (0–4 mm) could withstand loading and unloading cycles comparable to those experienced by conventional masonry mortars, highlighting the potential long-term durability of these lightweight recycled materials [24].

This study demonstrates the feasibility of producing a lightweight insulating mortar incorporating high volumes of recycled rigid PU foam and integrating it into an industrial production cycle. In the first part, the use of substantial quantities of recycled rigid PU foam (82.5–92.25% by volume)—to the best of the authors’ knowledge, not previously reported—as the primary lightweight aggregate is systematically evaluated. Relationships between rigid PU waste content and key performance indicators (density, mechanical strength, and thermal insulation) are established. In the second part, the development, installation, and evaluation of a prototype production system for thermal insulation mortars containing 92.25 vol.% PU particles is presented, with a demonstrated capacity of 1500 L/h (equivalent to 20 bags of 75 L).

## 2. Materials and Methods

### 2.1. PU Waste Materials

**Figure 2:**
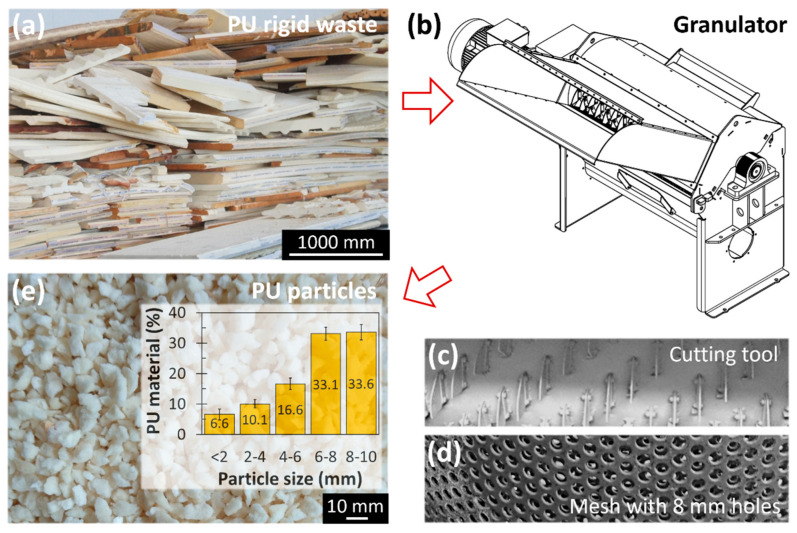
(**a**) Different rigid polyurethane foam waste materials, (**b**) granulator with (**c**) cutting tool and (**d**) mesh with 8 mm holes, and (**e**) material after granulating, with the particle size distribution displayed in the inset.

Rigid PU waste (Figure 2a) with a bulk density of 40 ± 2 kg/m^3^, sourced from the sandwich panel production process at Nicolaides & Kountouris Metal Company Ltd. (Nicosia, Cyprus), was used in the first part of this study to develop an optimized lightweight mortar. For the validation of the production system developed in the second part of the study, rigid PU waste from various sources (e.g., insulating materials, demolition/construction, refrigeration, packing material) with a bulk density of 40 ± 5 kg/m^3^ was employed. The granulation process was carried out using a high-speed heavy-duty granulator developed specifically for this study (Figure 2b), which was equipped with a rotary cutting tool (Figure 2c) and a stainless-steel mesh with 8 mm holes (Figure 2d). This setup enabled the reduction of PU waste into finer particles (Figure 2e). To assess the particle size distribution, three samples of 500 g each of the granulated PU material were sieved using a series of stainless-steel meshes with aperture sizes of 2, 4, 6, 8, and 10 mm. As shown in the inset of Figure 2e, approximately one-third of the particles were smaller than 6 mm, one-third ranged from 6 to 8 mm, and the remaining one-third exceeded 8 mm in size. The presence of particles larger than 8 mm, despite the use of an 8 mm mesh during granulation (Figure 2d), is attributed to the continuous feeding of the granulator. Under these conditions, larger PU fragments can be forced through the mesh due to mechanical compression and pressure exerted by the feed material.

### 2.2. Preparation of Mortar Mixtures

**Figure 3:**
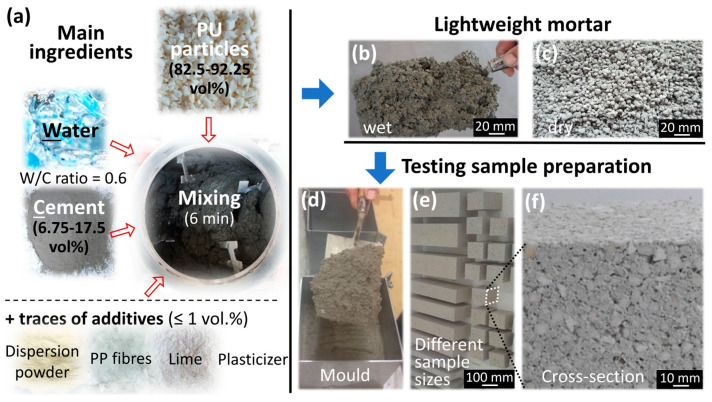
(**a**) Ingredients used for the fabrication of lightweight mortar, (**b**) wet and (**c**) dry mortar, (**d**) filling of a mold, (**e**) different testing sample sizes, and (**f**) cross-section of a dried lightweight mortar sample.

Eight different lightweight mortar mixtures were prepared by initially introducing 6.75–17.5 vol.% Portland cement (Vassiliko Cement Works Public Company Ltd., Nicosia, Cyprus) and water into a drum mixer at a fixed water-to-cement (W/C) ratio of 0.6, as illustrated in Figure 3a. This W/C ratio was selected because Gadea et al. [22], in their influential study on lightweight mortars incorporating recycled PU foam, reported that a reduction in the W/C ratio from approximately 0.8 to 0.6 was required to maintain good workability as the replacement of sand with PU foam increased from 0 to 100%. Granulated rigid PU foam waste (82.5–92.25 vol.%) was subsequently added as the primary lightweight aggregate, along with minor additives (~1 vol.%), including dispersion powder (C.G. Christofides Industrial Ltd., Nicosia, Cyprus) in all mixtures and additionally lime (Hellenic Mining Public Company Ltd., Nicosia, Cyprus), polypropylene (PP) fibers (Romfracht Srl, Caldararu, Romania) and plasticizer (Panaska Trading Co. Ltd., Nicosia, Cyprus) for mixtures containing PU waste above 91.25 vol.%. The components were thoroughly mixed until a homogeneous mortar was obtained (Figure 3b and Figure 3c show wet and dry mortar samples, respectively). The fresh mixtures were then cast into steel molds (Figure 3d) and demoulded after 24 h. Figure 3e displays various specimens after 28 days of curing, following the procedure described by Gomes et al. [25], while Figure 3f shows the cross-section of a 100 mm × 100 mm × 100 mm specimen, where larger PU particles embedded in the matrix are clearly visible.

### 2.3. Characterization Methods

Three specimens of each mortar mixture were prepared and subjected to testing in their hardened state after a standard curing period of 28 days. The testing procedures followed the guidelines specified in [26,27], which are part of the European standards for determining the physical and mechanical properties of hardened mortar. These procedures were supplemented with methodologies described in previous studies [19,20,22,23] to ensure consistency and comparability with existing literature. The hardened mortar specimens were assessed for three key properties: bulk density, compressive strength, and thermal conductivity.

The bulk density of the hardened lightweight mortar samples (100 mm × 100 mm × 100 mm) was assessed in accordance with EN 1015-10:1999 [26] by calculating the mass-to-volume ratio. For the performance assessment of the production system, skeletal density measurements of the final product (used mass: 40 ± 5 g) were also conducted using gas pycnometry with an Anton–Paar Ultrapyc 5000 device (Boynton Beach, FL, USA). High-purity (99.999%) helium (He) was used as the displacement gas to ensure accurate volume determination. The measurements were performed at a controlled temperature of ~20 °C with a He pressurization cycle ranging from 0.03 bar to 0.69 bar. Prior to the tests, the large sample cell (131.7 mL) was calibrated using a reference silicon sphere of known volume to ensure measurement accuracy. For each sample, the average skeletal density was calculated from five independent runs, exhibiting a variance of less than 0.1%, confirming high precision and repeatability.

Compressive strength testing was carried out in accordance with EN 1015-11:2000 [27], which specifies the method for determining the compressive strength of hardened concrete specimens. Cube-shaped samples with dimensions of 100 mm × 100 mm × 100 mm were used. Testing was performed using a fully automated INSTRON 5967 Universal Testing Machine (Norwood, MA, USA) equipped with a 30 kN load cell. Each specimen was carefully aligned so that the upper surface of the cube was in contact with the machine’s moving upper platen, while the lower surface rested on the fixed lower platen, ensuring uniform load distribution. The axial compressive load was applied at a constant displacement rate of 0.5 mm/min until failure, as recommended for low-density cementitious materials. The peak load at failure was recorded and used to calculate the compressive strength by dividing the load by the cross-sectional area of the specimen. All tests were conducted under controlled laboratory conditions (temperature: ~20 °C; relative humidity: ~50%).

Thermal conductivity (λ) of the lightweight mortar samples (dimensions: 300 mm × 300 mm × 50 mm) was measured using a FOX 200 Heat Flow Meter (TA Instruments, New Castle, DE, USA), in accordance with EN 12664:2001 [28]. This standard specifies the steady-state method for determining the thermal resistance and thermal conductivity of materials using a heat flow meter apparatus. During the test, a known temperature gradient is applied across the sample, and the resulting heat flux is measured, allowing accurate calculation of thermal conductivity. All measurements were conducted under controlled laboratory conditions to ensure consistency and reproducibility.

Both small- and full-scale fire tests were conducted on samples from the developed production system to assess the fire performance of the lightweight mortar. Small-scale ignitability tests were carried out in accordance with ISO 11925-2:2020 [29], while large-scale fire classification testing was performed following EN 13501-1 + A1:2010 [30], incorporating EN ISO 9239-1:2010 [31] and EN ISO 1716:2010 [32].

## 3. Results and Discussion

### 3.1. Development of Lightweight, Thermally Insulating Mortars Incorporating High Volumes of Recycled Rigid Polyurethane Foam

**Figure 4:**
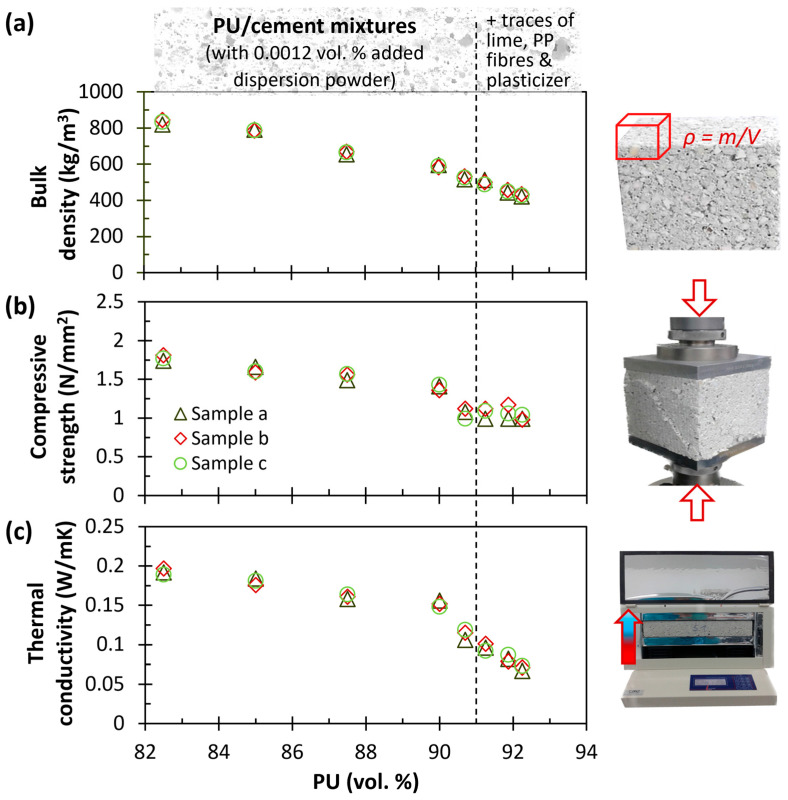
(**a**) Bulk density, (**b**) compressive strength, and (**c**) thermal conductivity results as a function of the amount of polyurethane (**left**), and samples/setups for the evaluation of these properties (**right**).

Figure 4 illustrates that bulk density (Figure 4a), compressive strength (Figure 4b), and thermal conductivity (Figure 4c) are primarily influenced by the proportion of recycled PU in the mix (the samples and experimental setups used to evaluate these properties are shown on the right). As anticipated, increasing the PU content from 82.5 to 92.25 vol.% resulted in a corresponding decrease in bulk density (from ~850 to 420 kg/m^3^), compressive strength (from ~1.8 to 1 N/mm^2^) and thermal conductivity (from ~0.19 to 0.07 W/m·K), due to the inherently lower mass, mechanical properties and thermal conductivity of the recycled PU compared to cement. This trend agrees with observations from other studies on lightweight mortar with recycled PU (although lower employed vol.%) rigid foam particles [22,23,24].

The PU particles (0–10 mm in size) used in this study are more comparable to those typically employed in the development of lightweight concrete, where particle sizes up to 10 mm [19] and 8–20 mm [20] have been reported. In contrast, previous studies on lightweight mortars have primarily used significantly smaller PU particles, ranging from 0–4 mm [22,24] or even 0–1 mm [23]. The use of larger PU particles in cement mortars offers several advantages and can help to explain the observed reduction in bulk density, compressive strength, and thermal conductivity. Their lower surface area-to-volume ratio and cellular structure enhance thermal insulation by trapping more air, thereby reducing thermal conductivity. Larger particles also contribute to lower mortar density, making them particularly suitable for non-load-bearing applications such as floor and roof insulation targeted in this study. Moreover, they absorb less water, which improves workability and reduces the need for additional admixtures. Their incorporation promotes a more uniform distribution of voids, which in turn improves resistance to environmental stress and enhances acoustic insulation. From a processing perspective, larger PU particles are more energy-efficient to produce and can be readily integrated into existing lightweight mix designs.

For each mixture, three measurements (labeled a, b, and c) were conducted, yielding highly consistent results. In the first five mixtures, consisting of 82.5–90.7 vol.% PU and 17.5–8.75 vol.% cement, only 0.0012 vol.% of dispersion powder was added. This additive improves adhesion between components, thereby improving the setting behavior and mechanical strength of the hardened mixtures [33]. In the three mixtures with higher PU content (91.25–92.25 vol.%) and lower cement content (8.75–6.75 vol.%), displayed on the right side of the dashed line in Figure 4, additional components were introduced: small amounts of (a) hydrated natural lime, serving as a hydraulic binder to improve mechanical strength and stability [34], (b) a plasticizer to enhance workability and usability [20], and (c) PP fibers to optimize mechanical performance and mitigate the formation of micro-cracks [35]. The optimized lightweight mortar mixture—comprising 92.25 vol.% PU, 6.75 vol.% cement (with a W/C ratio of 0.6), ~1 vol.% lime, 0.00055 vol.% dispersion powder, 0.00055 vol.% plasticizer, and 0.00028 vol.% PP fibers—exhibited excellent workability and leveling. It achieved a compressive strength of 1.0 N/mm^2^, a bulk density of 420 kg/m^3^, and a thermal conductivity of 0.07 W/m·K.

The compressive strength and bulk density values reported in this study are notably lower than those presented in references [22,23,24]. This difference can be attributed to substantial variations in mix design, including different ratios of cement, water, sand, and PU foam, as well as variations in PU particle size (0–4 mm in [22,24]; 0–1 mm in [23]). Furthermore, thermal conductivity values were not provided in any of these studies. Overall, the lower PU content in references [22,23,24] likely contributed to the higher density and compressive strength values observed in those works compared to our findings.

### 3.2. Design and Evaluation of Prototype Pilot-Scale Production System

**Figure 5:**
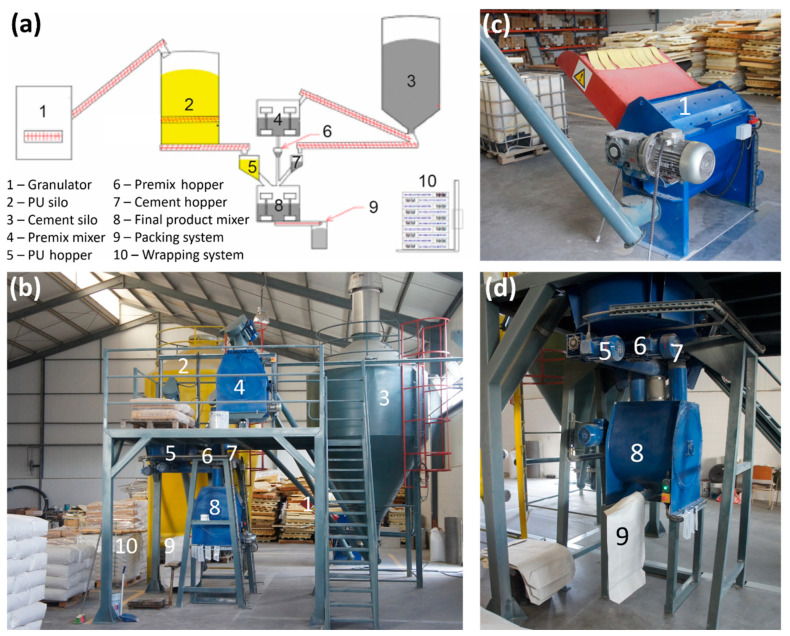
(**a**) Diagram of the production system for lightweight mortar and photos of (**b**) the production system, (**c**) the granulator, and (**d**) the hopper, mixing and packing system.

To scale up the production of the optimized lightweight mortar mixture developed in the above-described initial study, a prototype system was designed and installed, with a production capacity of 1500 L of insulating mortar per hour. Figure 5a and Figure 5b show the main components of the prototype system, including the granulator (1), PU silo (2), cement silo (3), premix mixer (4), PU hopper (5), premix hopper (6), cement hopper (7), final product mixer (8), packing system (9) and wrapping system (10).

A key component of the system is the specially designed granulator ((1); Figure 5c), which produces a uniform particle size (≤10 mm) of the rigid PU waste foam, as illustrated in the inset of Figure 2e. This granulated PU foam constitutes the main ingredient (92.25 vol.%) of the lightweight concrete mixture. Foam particles passing through an 8 mm mesh (Figure 2d) are conveyed via a screw conveyor to the granulator outlet and then transferred to the PU silo (capacity: 10 m^3^) for storage. At the base of the PU silo, a screw conveyor ensures uniform distribution of the granulated PU foam within the silo. To monitor the PU level, two sensors are installed and connected to a programmable logic controller (PLC), indicating the minimum and maximum fill levels. During system operation, an agitator operates continuously to prevent the PU foam from adhering to the silo walls.

Adjacent to the PU silo is the cement silo (3), also with a capacity of 10 m^3^ and equipped with a filling system. As for the PU silo, it includes two level sensors connected to the PLC for monitoring fill levels. The cement silo is linked to an air filtration system and two screw conveyors; one supplying cement to the premix mixer (4) and the other to the cement hopper (7).

In the premix mixer (4), a blend comprising cement, natural lime, plasticizer, dispersion powder, and PP fibers is prepared. Following mixing, the premix is transferred to the premix hopper (6) via a screw conveyor. The premix quantity used in the final mixture is regulated by a load cell installed on the premix hopper, while the cement dosage is controlled by a load cell on the cement hopper (7). An additional load cell on the premix mixer (4) ensures accurate premix dosing. In the final product mixer (8), cement, premix, and granulated PU foam are combined. The foam is delivered from the PU silo to a volumetric PU hopper (5) via a screw conveyor and then transported to the final product mixer using a horizontal screw conveyor. The volumetric hopper is equipped with two level sensors to indicate minimum and maximum fill levels. Accurate dosing of the PU foam is achieved by adjusting the screw conveyor’s transfer rate, controlled by the PLC. The final mortar product is packed into 75 L bags using the finished product screw conveyor (9). The filled bags are stacked on pallets, which are subsequently wrapped using an automated wrapping machine (10). The entire production process is fully automated and managed via a central PLC system. All operations are monitored and controlled from the main control cabinet. Figure 5d shows a photograph of the hopper, mixing, and packing systems.

**Figure 6:**
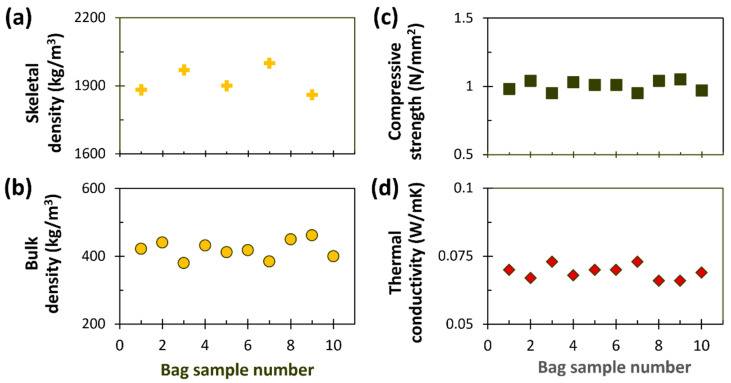
(**a**) Skeletal density, (**b**) bulk density, (**c**) compressive strength, and (**d**) thermal conductivity results for produced PU-cement samples.

For the validation of the developed production system, ten 75 L bag samples were randomly collected from different production batches of the final mortar product and subsequently analyzed. For the evaluation of bulk density, compressive strength, and thermal conductivity, the mortar was mixed with water, and test specimens were prepared and cured for 28 days before testing. Skeletal density was determined by using samples taken directly from five 75 L bags, representing different production batches. Figure 6 presents the average values per bag sample for skeletal density (Figure 6a), bulk density (Figure 6b), compressive strength (Figure 6c), and thermal conductivity (Figure 6d).

Skeletal density, also known as true density, is a fundamental material property representing the density of the solid phase (skeleton) of a material, excluding open pores and voids. It is determined by dividing the material’s mass by its skeletal volume, which is typically measured using He pycnometry. Unlike bulk density, which accounts for both the solid phase and pore volume, skeletal density provides insights exclusively into the solid phase, making it independent of particle size and geometry. By comparing skeletal density with bulk density, the material’s porosity (i.e., the fraction of pore volume relative to total volume) can be estimated. This parameter is particularly significant in fields such as construction and building materials, where porosity influences mechanical strength, durability, and permeability.

The skeletal densities of five production samples ranged from 1860 to 2050 kg/m^3^ (Figure 6a), with an average of 1930 kg/m^3^ across all samples. These differences can be attributed to the relatively small mass of the used dry mixture (40 ± 5 g), randomly taken from different 75 L bags, which may not have been perfectly homogeneous. For reference, two pure cement samples used in producing the composites were also measured, yielding similar skeletal densities of 3060 and 3070 kg/m^3^, respectively. The minimal variation between these values (~0.3%) suggests high measurement repeatability of the pycnometer. These values closely correspond to the skeletal density of Portland-type cement (3130 kg/m^3^) reported previously [36]. The lower skeletal densities of the composites (~37% lower on average than pure cements) are attributed to the significant amount of PU (92.25 vol.%) in the mixture. However, they remain approximately 1.8 times higher than the theoretical skeletal density of PU (~1050 kg/m^3^) [17]. In comparison, the average bulk density, based on ten specimens, was 420 kg/m^3^ (Figure 6b). Using these values, the total porosity was calculated as follows: porosity = [skeletal density − bulk density]/skeletal density. This yields a porosity of approximately 78%. This result aligns with expectations, as typical porosity values for conventional mortars (cement–sand mixtures) have been reported to range from approximately 17% to 40% for W/C ratios between 0.3 and 0.7 [37]. The significantly higher porosity observed here is attributed to the high content of rigid PU foam in the final mixtures (92.25 vol.%), as its low intrinsic density and open-cell structure promote increased overall porosity with rising PU content.

The values for bulk density (Figure 6b), compressive strength (Figure 6c), and thermal conductivity (Figure 6d) are generally consistent with those reported in the initial study and presented in Figure 4. However, higher standard deviations were observed, likely due to the use of rigid PU waste from various sources (e.g., insulating materials, demolition/construction, refrigeration, packing materials) with a density of 40 ± 5 kg/m^3^, compared to the more uniform PU waste used in the initial study from Nicolaides and Kountouris sandwich panels, which had a density of 40 ± 2 kg/m^3^.

The durability of the developed lightweight mortar was qualitatively assessed through a series of initial performance tests conducted during the product development phase. Although systematic, long-term monitoring under controlled curing and storage conditions has not yet been performed, observational data collected over a 12-month period indicate stable performance, with no evidence of mechanical or structural degradation. Compressive strength measurements remained consistent throughout the year, with only minor fluctuations within the bounds of experimental error. Accordingly, the reported compressive strength in this study includes a tolerance of ±5%. Density variations were limited to approximately 2–3%, which is typical for mortars incorporating lightweight aggregates and remains within the declared ±5% tolerance. Additionally, no signs of cracking, shrinkage, or mechanical failure were observed during internal testing over the 12-month period, further confirming the durability of the mortar under standard storage and handling conditions.

Small-scale ignitability tests revealed delayed ignition times and significantly reduced flame spread for the developed lightweight mortar compared to unbound PU foam. Results from large-scale fire classification testing further confirmed that the PU-based lightweight mortar, despite containing 92.25 vol.% of recycled PU foam, meets the criteria for A2fl–s1 classification (reaction to fire: A2fl; smoke production: s1), indicating a non-combustible flooring material with minimal smoke production. This enhanced fire performance—compared to rigid PU foam, which begins to decompose at relatively low temperatures (typically around 200 °C [38]) and thus poses a fire safety risk—can be attributed to the cementitious matrix. It acts as an effective thermal barrier, encapsulating the PU particles and protecting them from direct flame exposure.

**Figure 7:**
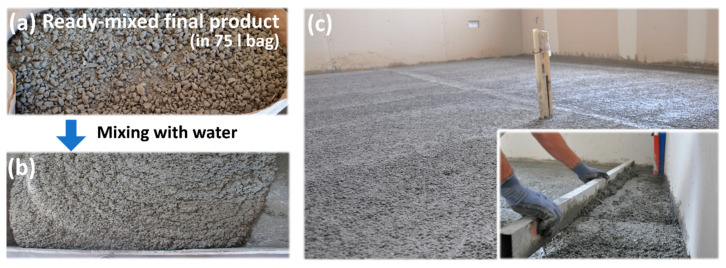
(**a**) Ready-mixed final lightweight mortar in a 75 L bag, (**b**) mixture after adding water, and (**c**) application example.

In summary, the lightweight mortar from the production system, comprising 92.25 vol.% recycled PU foam, exhibits a low density of 420 kg/m^3^ ± 5% and satisfactory mechanical performance, with a compressive strength of approximately 1 MPa (1 N/mm^2^ ± 5%). Its low thermal conductivity (λ = 0.07 W/m·K ± 5%) makes it particularly suitable for thermal insulation in existing roof and floor structures where structural load capacity is constrained. The combination of low density, adequate compressive strength, and enhanced insulation properties underscores the material’s potential for practical application in sustainable construction. The developed lightweight insulating mortar is supplied in 75 L paper bags (Figure 7a) and requires only the addition of 25 L of water prior to on-site application (Figure 7b). A representative example of its application is illustrated in Figure 7c.

## 4. Summary and Conclusions

This study investigated the feasibility of producing a lightweight insulating mortar using waste rigid polyurethane (PU) foam (≤10 mm particle size) as the primary aggregate, with the aim of integrating the process into an industrial production cycle. Various mortar formulations were initially evaluated to examine the relationship between mix design parameters and key performance indicators, namely density, mechanical strength, and thermal insulation. Preliminary results demonstrate that these properties are strongly influenced by the volume fraction of recycled PU incorporated into the mix.

The optimized formulation, containing 92.25 vol.% recycled PU foam, was successfully scaled from laboratory to industrial production. The resulting lightweight concrete demonstrated a low bulk density (420 kg/m^3^ ± 5%), adequate compressive strength (1 MPa ± 5%), and excellent thermal insulation (λ = 0.07 W/m·K ± 5%), making it particularly suitable for use in roof and floor applications where structural load capacity is limited. The process developed is scalable and enables the product to be packaged in 75 L paper bags, requiring only water for on-site application. Its properties also make it ideal for insulating floors and filling cavities. This approach presents a viable method for reducing PU waste and contributes to the development of eco-friendly building materials.

Future research will explore larger-scale production systems and the use of other plastic waste materials, such as polystyrene, to further support circular economy strategies in construction. Overall, the findings underscore the potential of PU-based recycled lightweight mortar to reduce environmental impact and promote sustainable building practices globally.

## Data Availability

The raw data supporting the conclusions of this article will be made available by the authors on request.

## References

[1] Nayanathara Thathsarani Pilapitiya P.G.C., Ratnayake A.S. (2024). The world of plastic waste: A review. Clean. Mater..

[2] Plastics Europe (2023). Plastics—The Fast Facts 2023.

[3] Banik J., Chakraborty D., Rizwan M., Shaik A.H., Chandan M.R. (2023). Review on disposal, recycling and management of waste polyurethane foams: A way ahead. Waste Manag. Res..

[4] Rossignolo G., Malucelli G., Lorenzetti A. (2023). Recycling of polyurethanes: Where we are and where we are going. Green Chem..

[5] Dokl M., Copot A., Krajnc D., Fan Y.V., Vujanović A., Aviso K.B., Tan R.R., Kravanja Z., Čuček L. (2024). Global projections of plastic use, end-of-life fate and potential changes in consumption, reduction, recycling and replacement with bioplastics to 2050. Sustain. Prod. Consum..

[6] Janssens V. (2022). Plastics—The Facts 2022.

[7] Kemona A., Piotrowska M. (2020). Polyurethane recycling and disposal: Methods and prospects. Polymers.

[8] Gadhave R.V., Srivastava S., Mahanwar P.A., Gadekar P.T. (2019). Recycling and Disposal Methods for Polyurethane Wastes: A Review. Open J. Polym. Chem..

[9] Jethy B., Paul S., Das S.K., Adesina A., Mustakim S.M. (2022). Critical review on the evolution, properties, and utilization of plasticwastes for construction applications. J. Mater. Cycles Waste Manag..

[10] Alqahtani F.K., Zafar I. (2021). Plastic-based sustainable synthetic aggregate in Green Lightweight concrete—A review. Constr. Build. Mater..

[11] Ahmed H.U., Faraj R.H., Hilal N., Mohammed A.A., Sherwani A.F.H. (2021). Use of recycled fibers in concrete composites: A systematic comprehensive review. Compos. Part B Eng..

[12] Adesina A. (2021). Overview of the influence of waste materials on the thermal conductivity of cementitious composites. Clean. Eng. Technol..

[13] Roobankumar R., SenthilPandian M. (2024). A review of utilization of waste polyurethane foam as lightweight aggregate in concrete. Heliyon.

[14] Mistry M., Prajapati V., Dholakiya B.Z. (2024). Redefining Construction: An In-Depth Review of Sustainable Polyurethane Applications.

[15] Somarathna H.M.C.C., Raman S.N., Mohotti D., Mutalib A.A., Badri K.H. (2018). The use of polyurethane for structural and infrastructural engineering applications: A state-of-the-art review. Constr. Build. Mater..

[16] Gómez-Rojo R., Alameda L., Rodríguez Á., Calderón V., Gutiérrez-González S. (2019). Characterization of polyurethane foam waste for reuse in eco-efficient building materials. Polymers.

[17] Nodehi M. (2022). Epoxy, polyester and vinyl ester based polymer concrete: A review. Innov. Infrastruct. Solut..

[18] Saikia N., De Brito J. (2012). Use of plastic waste as aggregate in cement mortar and concrete preparation: A review. Constr. Build. Mater..

[19] Mounanga P., Gbongbon W., Poullain P., Turcry P. (2008). Proportioning and characterization of lightweight concrete mixtures made with rigid polyurethane foam wastes. Cem. Concr. Compos..

[20] Ben Fraj A., Kismi M., Mounanga P. (2010). Valorization of coarse rigid polyurethane foam waste in lightweight aggregate concrete. Constr. Build. Mater..

[21] Rooban Kumar R., Senthil Pandian M. (2024). Utilisation of solid waste polyurethane foam as coarse aggregate in concrete: An experimental study with ecological and cost assessment. J. Mater. Cycles Waste Manag..

[22] Gadea J., Rodríguez A., Campos P.L., Garabito J., Calderón V. (2010). Lightweight mortar made with recycled polyurethane foam. Cem. Concr. Compos..

[23] Junco C., Gadea J., Rodríguez A., Gutiérrez-González S., Calderón V. (2012). Durability of lightweight masonry mortars made with white recycled polyurethane foam. Cem. Concr. Compos..

[24] Junco C., Rodríguez A., Calderón V., Muñoz-Rupérez C., Gutiérrez-González S. (2018). Fatigue durability test of mortars incorporating polyurethane foam wastes. Constr. Build. Mater..

[25] Gomes M.G., Flores-Colen I., da Silva F., Pedroso M. (2018). Thermal conductivity measurement of thermal insulating mortars with EPS and silica aerogel by steady-state and transient methods. Constr. Build. Mater..

[26] (1999). Methods of Test for Mortar for Masonry—Part 10. Determination of Dry Bulk Density of Hardened Mortar 1999.

[27] (2000). Methods of Test for Mortar for Masonry—Part 11. Determination of Flexural and Compressive Strength of Hardened Mortar 2000.

[28] (2001). Thermal performance of building materials and products—Determination of thermal resistance by means of guarded hot plate and heat flow meter methods—Dry and moist products of medium and low thermal resistance 2001.

[29] (2020). Reaction to Fire Tests—Ignitability of Products Subjected to Direct Impingement of Flame—Part 2: Single-Flame Source Test.

[30] (2010). Fire classification of Construction Products and Building Elements—Part 1: Classification Using Data from Reaction to Fire Tests.

[31] (2010). Reaction to Fire Tests for Floorings—Part 1: Determination of Burning Behaviour Using a Radiant Heat Source.

[32] (2010). Determination of the Gross Heat of Combustion.

[33] Li H., Gu L., Dong B., Chen Q., Xu C., Yang X., Wang W. (2020). Improvements in setting behavior and strengths of cement paste/mortar with EVA redispersible powder using C-S-Hs-PCE. Constr. Build. Mater..

[34] Emmanuel O.U., Kuqo A., Mai C. (2021). Non-conventional mineral binder-bonded lignocellulosic composite materials: A review. BioResources.

[35] Yang S., Yue X., Liu X., Tong Y. (2015). Properties of self-compacting lightweight concrete containing recycled plastic particles. Constr. Build. Mater..

[36] Park S., Kang M.C., Oinam Y., Amoozegar A., Pyo S. (2022). Measurement of skeletal density and porosity of construction materials using a new proposed vacuum pycnometer. Meas. J. Int. Meas. Confed..

[37] He R., Ma H., Hafiz R.B., Fu C., Jin X., He J. (2018). Determining porosity and pore network connectivity of cement-based materials by a modified non-contact electrical resistivity measurement: Experiment and theory. Mater. Des..

[38] Mizera K., Sałasińska K., Borucka M., Przybysz J., Gajek A. (2025). Analysis of the Thermal Decomposition and Flammability of Polyurethane Materials Used in Building Insulation and in the Automotive Industry. Fire Mater..

